# Self-healing CD30- T-clonal proliferation of the tongue: report of an extremely rare case

**DOI:** 10.1186/s12903-019-0875-5

**Published:** 2019-08-15

**Authors:** Giacomo Setti, Eugenia Martella, Cristina Mancini, Paolo Vescovi, Cristina Magnoni, Pierantonio Bellini, Ilaria Giovannacci, Marco Meleti

**Affiliations:** 1 Dipartimento di Medicina e Chirurgia, Reparto di Patologia e Chirurgia Orale Laser, Centro Universitario di Odontoiatria, Università di Parma, via Gramsci 14, 43126 Parma, Italy; 2 Dipartimento Diagnostico, Unità Operativa Complessa di Anatomia e Istologia Patologica, Azienda Ospedaliero Universitaria di Parma, via Gramsci, 14, 43126 Parma, Italy; 3 Dipartimento Chi.Mo.Mo., Struttura Complessa di Dermatologia, Università di Modena e Reggio Emilia, Largo del Pozzo 71, 41125 Modena, Italy; 4 Dipartimento Chi.Mo.Mo., Struttura Complessa di Odontoiatria e Chirurgia Oro-maxillofacciale, Università di Modena e Reggio Emilia, Largo del Pozzo 71, 41125 Modena, Italy

**Keywords:** Oral pathology, Oral medicine, Tugse, Lymphoma, CD30-

## Abstract

**Background:**

The etiology of traumatic ulcerative granulomas with stromal eosinophilia (TUGSE) is not clear, traumatic irritation having advocated as the most likely cause. TUGSEs are typically self-limiting slow-healing lesions of the oral mucosa with unclear pathogenesis, commonly manifesting as a rapidly developing, long-lasting ulcer.

**Case presentation:**

Here we report a controversial case of a self-healing lesion of the tongue in a 57 year-old woman. A clonal T-cell proliferation and CD30 negative immunohistochemical (IHC) profile could be documented.

**Discussion and conclusion:**

In view of the very peculiar clinical and histological features, a retrospective diagnosis of a TUGSE with scarce eosinophilic infiltrate (possibly in regression), displaying CD30- T-clonal proliferation was eventually rendered.

The patient did not report signs of recurrence after a 3-year follow-up period.

## Background

Self-healing CD30- T-clonal proliferations of the oral cavity are exceedingly rare, existing only sporadic reports [1]. Mucosal lesions having such a peculiar immunohistochemical (IHC) pattern could underlay the heterogeneous histologic aspect of traumatic ulcerative granulomas with stromal eosinophilia (TUGSE). TUGSEs are typically self-limiting slow-healing lesions of the oral mucosa with unclear pathogenesis, commonly manifesting as a rapidly developing ulcer. Lesions usually persist for weeks or months with elevated or rolled borders [2].

The etiology of TUGSE is not clear, traumatic irritation having advocated as the most likely cause [3]). Clinically, they may mimic squamous cell carcinoma (SCC), warranting a biopsy or excision. TUGSEs are most often found on the tongue but they can occur in other areas, including gingiva, buccal and vestibular mucosa. Rapid healing after a biopsy is typical.

Different studies have demonstrated expression of CD30 and T cell–lineage antigens, suggesting that atypical mononuclear cells are of T-cell origin. Moreover, molecular evidence of T-cell clonality within TUGSE was described, raising the possibility that a subgroup of these lesions could be classified as low-grade CD30+ T-cell lymphoproliferative disorders [4]. T-cell clonality has been described in various benign and reactive conditions, and its presence does not necessarily indicate a T-cell malignancy. Additionally, some authors have hypothesized that TUGSE may be the oral counterpart of primary cutaneous CD30+ lymphoproliferative disorder [5].

Here we report a controversial case of a self-healing lesion of the tongue with clonal T-cell proliferation and CD30- IHC profile.

## Case presentation

**Fig. 1:**
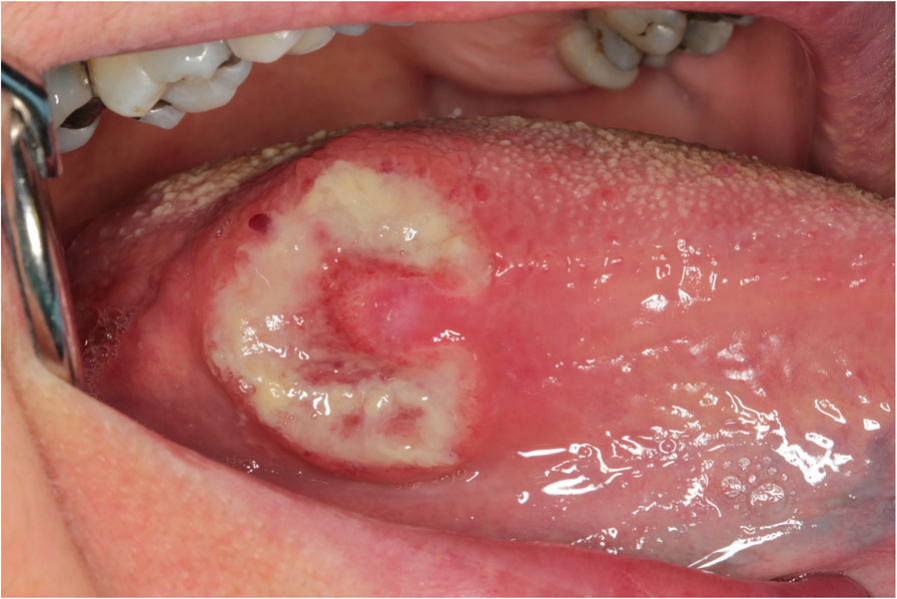
Lesion clinical appearance. Round, nodular, ulcerated, slightly exophytic lesion of the right posterior border of the tongue, in proximity of tongue ventral portion

A 57 year-old woman was referred to the Unit of Oral Medicine and Laser Surgery of the University of Parma, Italy, because of a 15 days standing, painful lesion on the right border of the tongue. Clinical investigation revealed the presence of a round, nodular, ulcerated, slightly exophytic lesion of the right posterior border of the tongue, in proximity of the ventral portion (Fig. 1). Lesion measured approximately 3 cm in diameter. According to the patient, no mechanical or thermal trauma had occurred.

Swelling of the affected part of the tongue was appreciable on palpation; no mobility impairment or presumptive infiltration in the deeper muscular layers were evident. Gauze swiping did not remove the fibrin-like material from the surface of the lesion.

Neck palpation did not allow the disclosure of enlarged cervical, supraclavicular, occipital and pre-auricular nodes.

Medical history was negative. Patient was non-smoker and she referred no alcohol consumption.

Differential diagnosis included: SCC, bacterial infections (e.g.: tuberculosis), lymphoproliferative disorder, chronic eosinophilic ulceration and possible trauma or chronic mutilation of which patient was not aware.

**Fig. 2:**
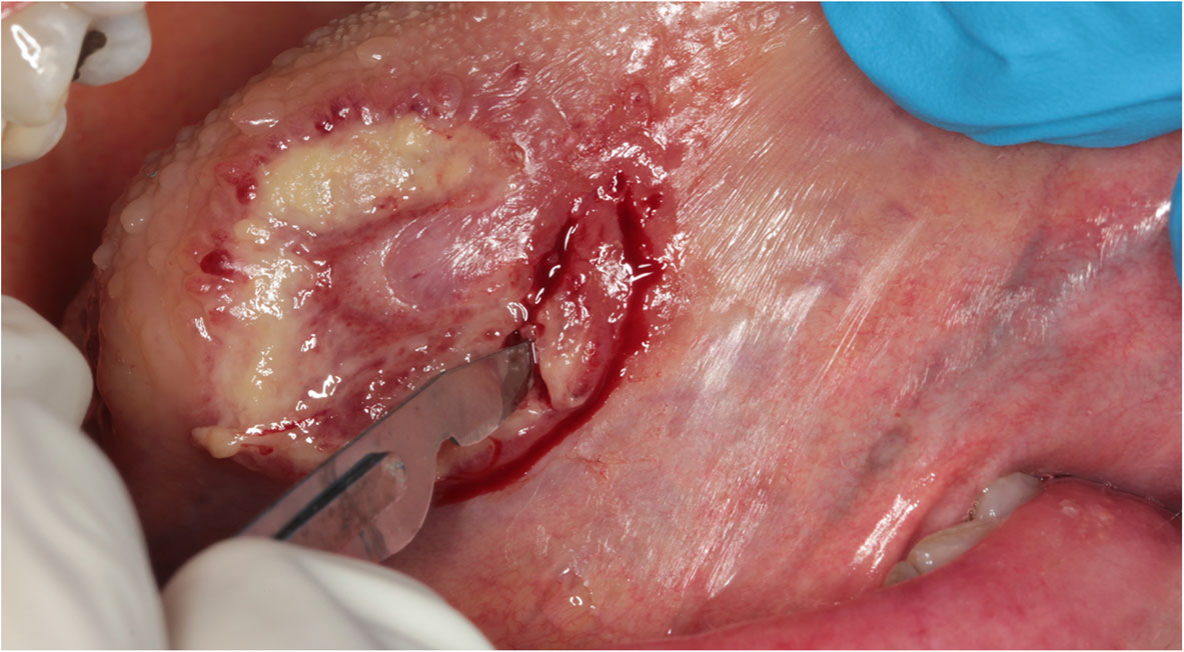
Multiple incisional biopsies surgical procedure

Two incisional biopsies were taken in the most suspicious parts (Fig. 2).

Further work-up included complete blood tests, chest x-rays, neck nodes and spleen ultrasonography (US), and magnetic resonance imaging of the head and neck region.

**Fig. 3:**
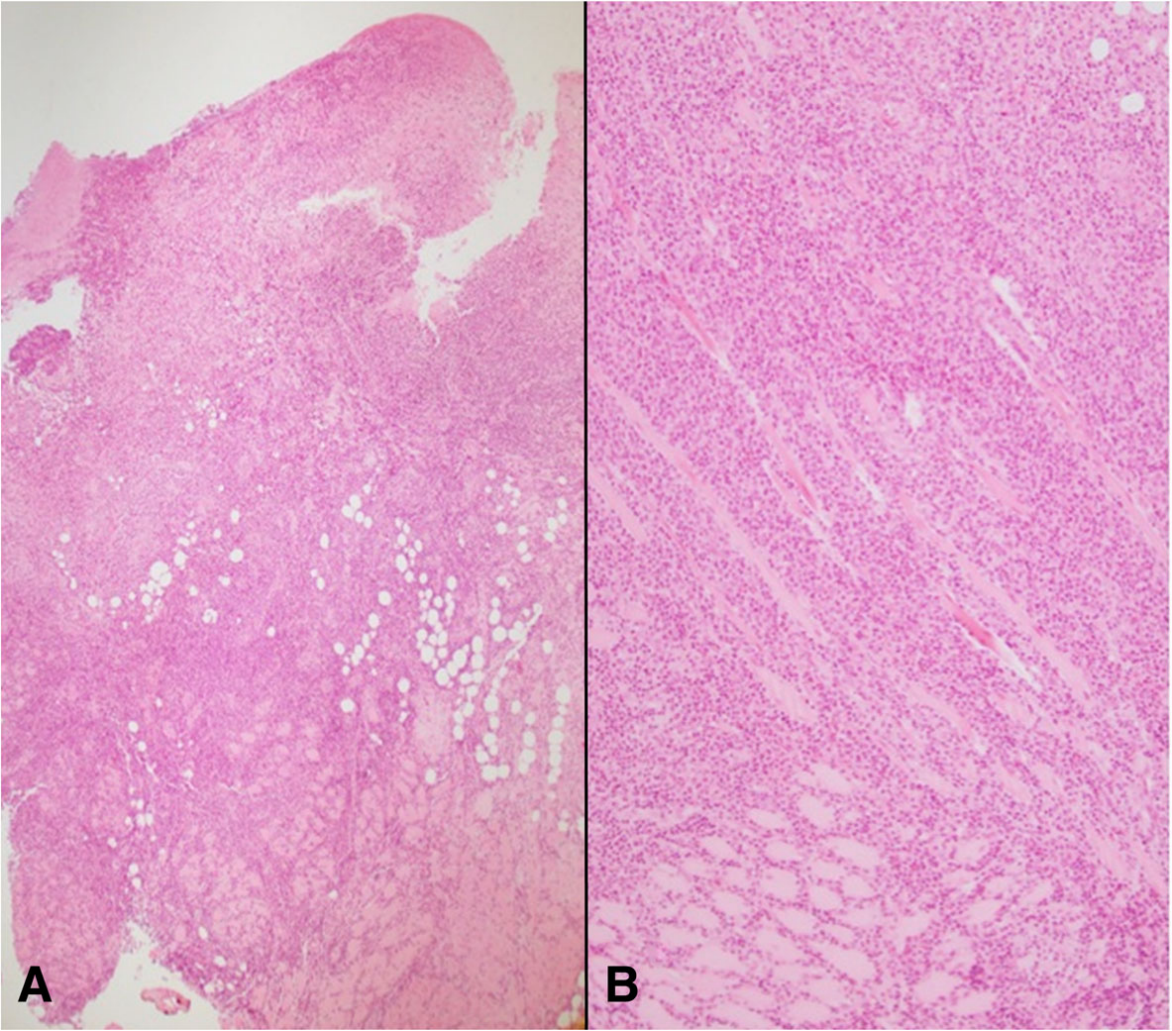
Histological examination, hematoxylin and eosin A-4x and B-10x. A polymorphic lymphocytic infiltrate with slight eosinophilia is appreciable. Epithelium presented ulceration with signs of pseudoepitheliomatous hyperplasia

Histological features included a polymorphic lymphocytic infiltrate with slight eosinophilia (Fig. 3). Epithelium presented ulceration with signs of pseudoepitheliomatous hyperplasia.

**Fig. 4:**
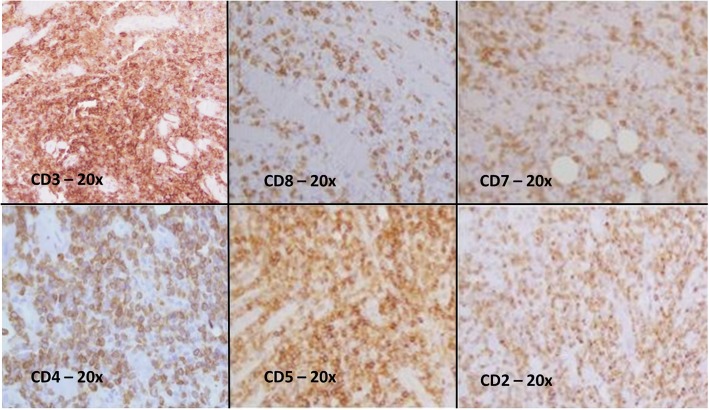
IHC analysis showed a lymphoid infiltrate with the following characterization: CD3+, CD2+, CD4+, CD8-, CD5+, CD7-

**Fig. 5:**
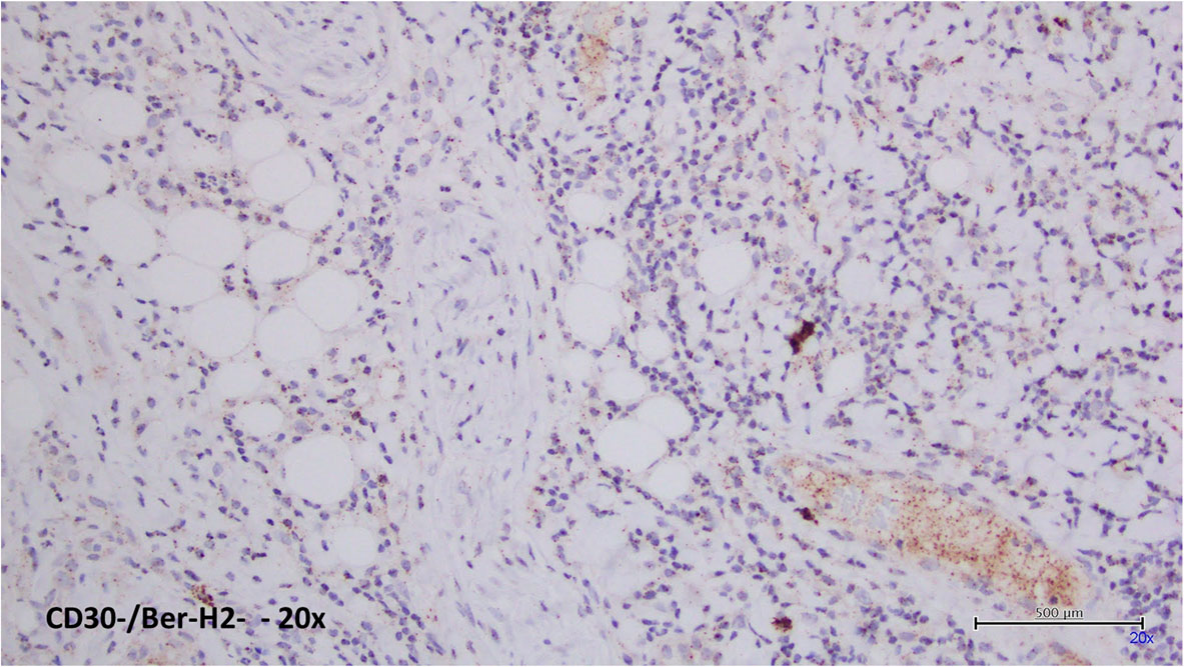
IHC analysis showed a lymphoid infiltrate with CD30−/Ber-H2- characterization (20x)

IHC analysis showed a lymphoid infiltrate with the following characterization: CD3+, CD2+, CD4+, CD8-, CD5+, CD7-, CD56-, Perforin -, CD30−/Ber-H2-, CD20-, CD1A-.(Figs. 4 and 5) Epstein-Barr Virus (EBV) investigation (Fluorescein-Conjugated Epstein-Barr Virus staining, EBER) was negative. Proliferation index Ki67 was 50%. Cytokeratin pool investigation was negative.

**Fig. 6:**
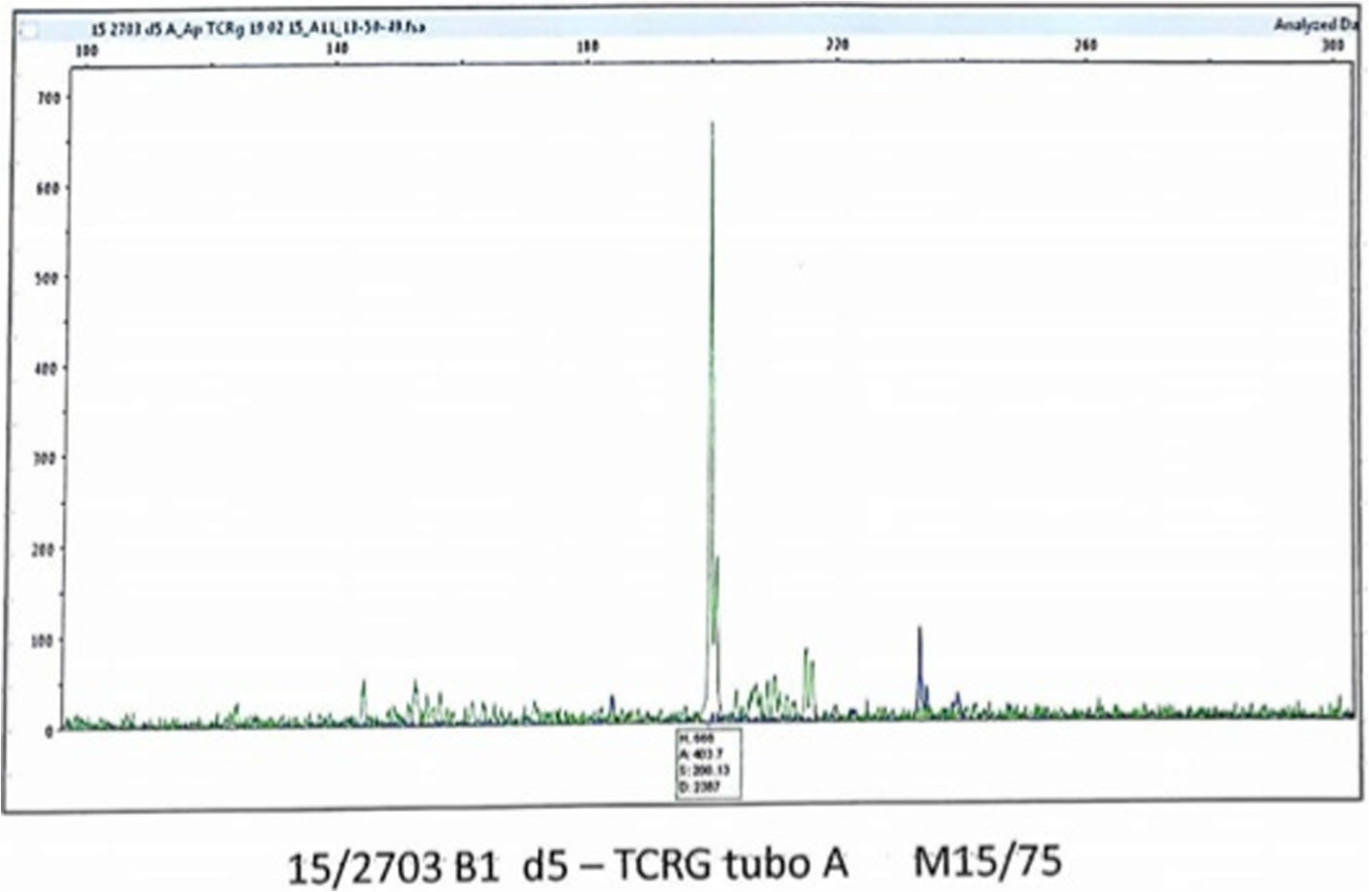
Molecular analysis - Monoclonality of TCRγ analysis: rearrangement was positive, showing monoclonal proliferation

Multiplexed polymerase chain reaction (PCR) revealed the presence of a monoclonal pattern and TCRγ (T cell receptor gamma) gene rearrangement (region Vg1–8/Vg10 - Jg 1.1/2.1 - Jg 1.3/2.3) (Fig. 6).

**Fig. 7:**
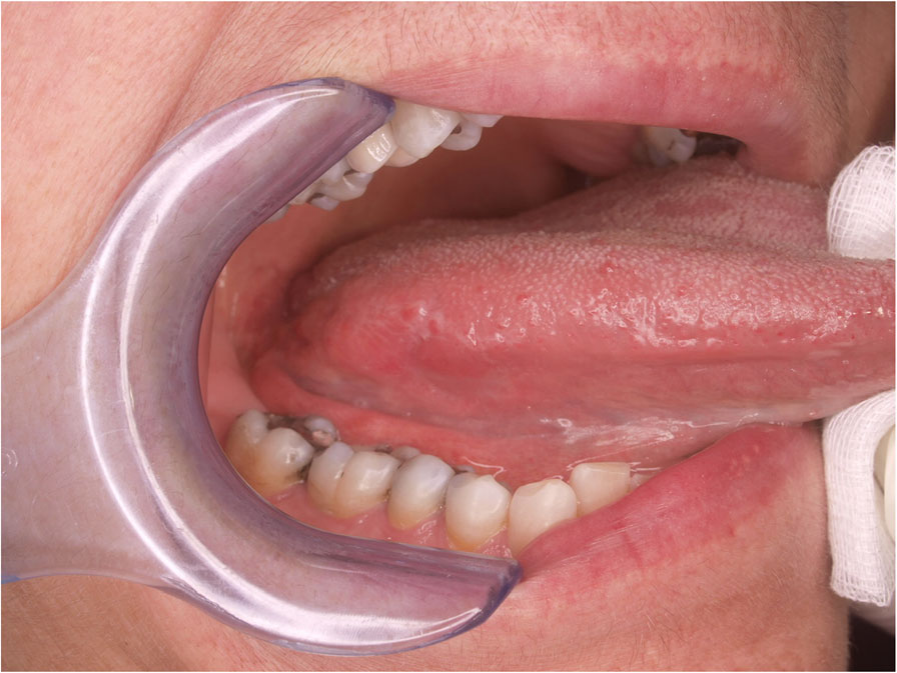
Complete self-healing after 15 days recall

Imaging and laboratory work-up was negative. In contrast with the seriousness of the clinical appearance, complete remission with mucosal healing spontaneously occurred in 15 days (Fig. 7).

In view of the very peculiar clinical and histological features, a retrospective diagnosis of a TUGSE with scarce eosinophilic infiltrate (possibly in regression), displaying CD30- T-clonal proliferation was eventually rendered.

**Fig. 8:**
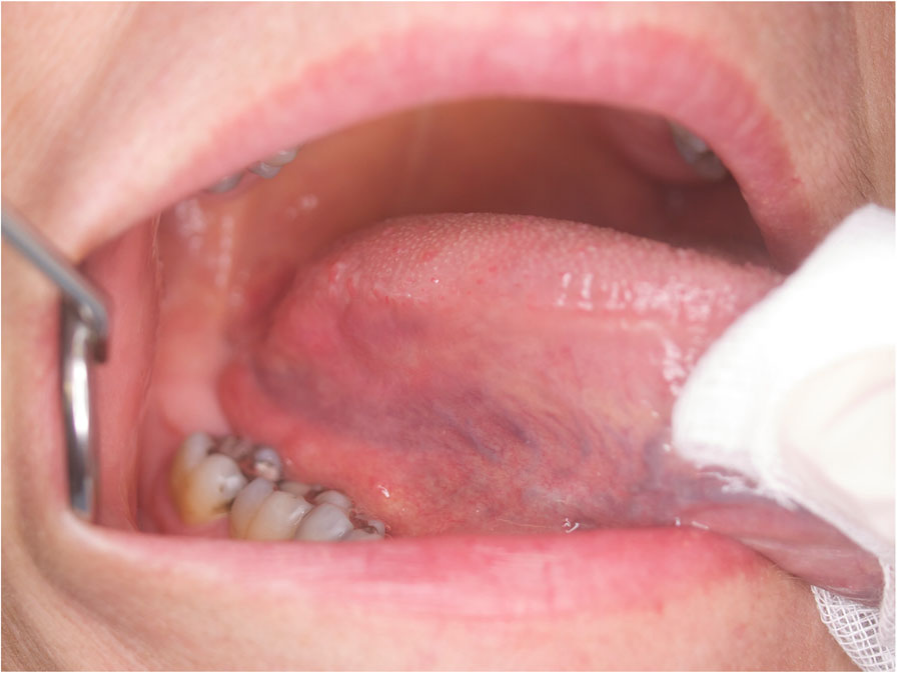
Tongue control after 3 years. No sign or symptoms of recurrence were registered during follow-up

The patient did not report signs of recurrence after a 3-year follow-up period (Fig. 8).

## Discussion and conclusion

Benign and malignant lymphoproliferative disorders may mimic either common oral conditions, including mucosal burns (chemical or thermal), traumatic ulcerations, SCC and rare lesions such as EBV+ mucosal ulcer and TUGSE. Such lesions therefore need an extremely accurate differential diagnosis.

Eosinophilic ulcer is a rare benign, reactive and self-limiting entity, first described clinically in infants by Riga in 1881 and histologically by Fede in 1890 (Riga-Fede disease) [6]. Described in adult by Popoff in 1956, it was identified as a distinct entity by Shapiro and Juhlin in 1970 [7]. The lesion has been indicated through different names, including traumatic eosinophilic granuloma of tongue, traumatic granuloma, atypical histiocytic granuloma and TUGSE [3]. A bimodal age distribution, with the first peak occurring at early childhood and the second during the 6th and 7th decade of life, has been reported [8]. No significant gender predilection has been highlighted, although some authors have evidenced a slightly higher female prevalence [9, 10].

The etiology of TUGSE is mostly unknown, although mechanical trauma has been considered as a typical trigger [3]. Some authors suggested that trauma could be a contributing factor in the development of ulcers, by leading to microbial infections followed by intense inflammatory response [11]. Others authors stated that cell-mediated immunity [12], CD30 lymphoproliferative disorders [4, 5, 13], mast cell- eosinophil interaction [14] and stress [15] may play a primary role in the pathogenesis of the lesion. Delayed healing of TUGSE lesions has been reported to be associated with the lack of secretion of transforming growth factor-α (TGF-α) and TGF-β by eosinophils infiltrating the lesions.

TUGSE commonly involves the tongue; other affected sites are the buccal and, labial mucosa the floor of the mouth the palate and vestibule. Lymphadenopathy can occur but it is extremely rare [12]. Clinically, the lesion appears as a singular entity. A case of multiple lesions has been reported in a young man affected by Riley-Day syndrome [7].

The lesion usually presents with raised, indurated borders and a yellow fibrinous floor. Because of its clinical appearance as well as the frequent long-standing duration, a suspicion of malignancy is usually included in the differential diagnosis.

Form the histologic point of view, under an ulcerated mucosa, a poorly formed granulation tissue showing an increased number of capillaries with prominent endothelial cells is usually observed. A dense submucosal polymorphous inflammatory infiltrate occasionally involving the overlying epithelium is frequently present. The infiltrate have a tendency to extend to the deeper soft tissues, (e.g. muscle fibers and salivary glands). The inflammatory infiltrate is composed of small round lymphocytes, abundant polymorphonuclear eosinophils and other inflammatory cells (neutrophils, plasma cells and histiocytes) [5, 10, 13, 14].

Large mononuclear atypic cells, intermingled in the inflammatory infiltrate are also frequently observed, occasionally (24% cases) with T-cell receptor gene rearrangement [1].

Many treatments are reported in literature, including wait-and-see, antibiotics, topical, intra-lesion and/or systemic corticosteroids, curettage, cryosurgery and surgical excision/incision [7]. Furthermore, it has been suggested to remove sources of local irritation (e.g. sharp teeth). Spontaneous healing frequently occurs within 30 days, even if cases of 8-month standing lesions have been reported [16].

Differential diagnosis of TUGSE may include mucosal burns (chemical or thermal), traumatic ulcer, malignancies (e.g. SCC or B-cell lymphoma), major aphthae (Sutton’s disease), Bechet’s syndrome, oral tuberculosis, primary syphilis or the recently documented Epstein-Barr virus positive muco-cutaneous ulcer (EBV MCU). EBV MCU has been described in the oral cavity, gastrointestinal tract, and skin. Such lesion, represent a unique, indolent form of EBV-driven B-cell lymphoproliferative disorder that presents as isolated, well-delineated ulcers. Documented cases have occurred in patients on immunosuppressive medication, in the elderly with age related immune-senescence and in immunodeficiency caused by HIV infection.

In the present case, the site (border of the tongue), the ulcerative/nodular clinical pattern, as well as the absence of causing factors, as reported in the anamnesis, were highly suggestive of a malignant lesion. Such features justified the immediate taking of multiple biopsies.

Apart from the extremely rare histopathologic and IHC pattern, the peculiarity of the case reported here lays on the surprising and unexpected benign clinical behavior, in contrast to a diagnosis, at least in the early phases, suggesting a T-cell lymphoproliferative disease.

In cases of diagnostic doubts it is always strongly recommended to carefully search for other features typical of malignant lymphoma. Features like abundant atypical cells infiltrate, significant mitotic activity, and infiltration into blood vessel walls should be investigated. In some cases, however, the diagnosis may be difficult to reach with confidence on the basis of histologic and IHC findings alone. For example, low-grade lymphoma is usually recognized only retrospectively, if lesions recur several times, spread to other areas, or they develop more pronounced microscopic malignant features [2].

CD30 is a transmembrane protein of the tumor necrosis factor (TNF) family; is a lymphocyte activation antigen important in diagnosis of classic Hodgkin’s lymphoma, anaplastic large cell lymphoma, embryonal carcinoma and primary cutaneous CD30+ T-cell lymphoproliferative disorders.

CD30+ cells scattered or clustered infiltrates were found in 5 out of 12 (42%), 11 out of 19 (58%), and 26 out of 37 TUGSE lesions (70%), as reported by Hirshberg et al., Fonseca et al. and Salisbury et al., respectively [1, 2, 10]. These CD30+ cells in TUGSE lesions include large mononuclear cells and small reactive T cells [17].

CD30- T-clonal oral lesions are very uncommon and, to the best of our knowledge, only two cases have been documented in details [1]. In both cases no recurrences were reported in a follow-up of 2 and 26 months. Negativity to CD30 is in contrast with the usual pattern of primary mucosal lymphoproliferative disorders which are CD30 + .

Immunohistochemistry for CD30 protein and PCR analysis for the TCR chain gene has shown that some atypical TUGSE display CD30 cells and they have a monoclonal rearrangement of the TCRγ chain gene [1, 2]. However, negative expression of anaplastic lymphoma kinase protein, a marker associated with large cell CD30+ lymphoma, supported the reactive nature of the lesions.

In the present case, TCRγ rearrangement was positive, showing monoclonal proliferation. However, no further evidences suggested a malignant behavior. Clonal proliferation was also described in CD30- lesions, with no influence on disease progression [1].

In view of the very heterogeneous histological pattern and clinical behavior of TUGSE and TUGSE-like lesions, careful management of patients is recommended. In cases of doubts, further work-up and close follow-up examinations should be performed in order to confirm or exclude the presence of malignant disorders such as lymphomas [18].

TUGSE should be regarded as a benign, reactive lesion rather than a T-cell lymphoproliferative disorder. Similarly to the case described here, TUGSEs with clonal TCR gene rearrangements reported in the literature had a benign clinical course. Our patient did not present further oral, systemic or skin lesions during a 3-year follow-up.

Detection of a dominant clone of a T-cell population as a single feature in a lymphoproliferative process is insufficient to diagnose a T-cell lymphoma. Because spontaneous regression of T-cell lymphoma is a recognized phenomenon, in the presence of monoclonality, a lymphoma work-up should always be undertaken.

In conclusion, without clinical, morphologic, or other supporting evidence, T-cell lymphoproliferative disorder diagnosis is not warranted. Overtreatment should be avoided.

## Data Availability

Not applicable.

## Abbreviations

EBER: Epstein Barr Encoding Region
EBV MCU: Epstein Barr virus muco-cutaneous ulcer
EBV: Epstein-Barr virus
IHC: Immunohistochemical
PCR: Polymerase chain reaction
SCC: Squamous cell carcinoma
TCRγ: T cell receptor gamma
TGF-α and –β: Transforming growth factor α and β
TNF: Tumor necrosis factor
TUGSE: Traumatic ulcerative granuloma with stromal eosinophilia
US: Ultrasonography
